# One-phase Split-bolus CT Urography – a Novel Approach To Reduce Radiation Dose in Diagnostics of Congenital Anomalies of Kidneys and Urinary Tract in Children

**DOI:** 10.34763/devperiodmed.20172104.402407

**Published:** 2018-01-02

**Authors:** Przemysław Bombiński, Michał Brzewski, Stanisław Warchoł, Marek Gołębiowski

**Affiliations:** 1 Department of Pediatric Radiology, Medical University of Warsaw, Warsaw, Poland; 2 Department of Pediatric Surgery and Urology, Medical University of Warsaw, Warsaw, Poland; 3 1st Department of Clinical Radiology, Medical University of Warsaw, Warsaw, Poland

**Keywords:** child, diagnostic techniques, - urological, Multidetector Computed Tomography, dziecko, metody diagnostyczne-urologiczne, wielorzędowa tomografia komputerowa

## Abstract

**Background:**

Low-dose CT Urography (LD-CTU) has become a standard procedure in urinary tract abnormalities in children, especially when MR Urography is not available. Standard one-phase CTU is performed in excretory phase. There is also a split-bolus technique, which combines two or even three phases during one scan and provides more clinical information without higher radiation exposure. It can be implemented for congenital anomalies of kidneys and urinary tract (CAKUT) in children, however, this application is not deeply discussed in scientific literature. Aim of this publication is to present the protocol and determine the role of LD-CTU in diagnostic imaging of CAKUT in children.

**Material and methods:**

Close to 300 CTUs in children were performed as a standard of care during the last 6 years in our Department. Diagnostic accuracy in suspected CAKUT was analyzed, depending on applied protocol − standard excretory CTU, multiphase CTU and two different one-phase split-bolus CTU protocols.

**Results:**

Visualization of the urinary tract was adequate in all study protocols. However, more clinically significant information was received in vascular-excretory protocol, including vascular and renal anatomy. Radiation exposure was similar or even lower than in other study protocols.

**Conclusions:**

One-phase split-bolus CTU protocol is a novel approach in low-dose diagnostic imaging of CAKUT in children. Combination of vascular and excretory phases has been shown as very effective technique, especially in comprehensive anatomical assessment of the abnormality and qualification to surgical intervention.

## Streszczenie

**Wstęp:**

Niskodawkowa Urografia TK (Uro-TK) stanowi podstawową metodę obrazowania wad wrodzonych układu moczowego u dzieci, zwłaszcza gdy niedostępna jest urografia MR. Standardowa jednofazowa Uro-TK wykonywana jest w fazie wydalniczej. Nowa technika podzielonego podania bolusa środka kontrastowego łączy dwie lub nawet trzy fazy w czasie pojedynczego skanowania, umożliwiając uzyskanie więcej danych bez narażenia pacjenta na wyższą dawkę promieniowania. Mimo że metoda może być wykorzystana w diagnostyce wad wrodzonych układu moczowego u dzieci, to w dostępnej literaturze brakuje doniesień dotyczących jej zastosowania w tej grupie pacjentów. Celem pracy jest przedstawienie protokołu badania i określenie miejsca niskodawkowej Uro-TK w diagnostyce wad wrodzonych układu moczowego u dzieci

**Materiał i metody:**

Na podstawie wykonanych w naszym Zakładzie w ciągu ostatnich 6 lat blisko 300 badań Uro-TK u dzieci, oceniono skuteczność diagnostyczną Uro-TK wykonanego wielofazowo oraz jednofazowo - w fazie wydalniczej oraz w dwóch różnych protokołach wykorzystujących podzielony bolus środka kontrastowego.

**Wyniki:**

Wszystkie poddane analizie protokoły badania umożliwiły odpowiednie zobrazowanie dróg moczowych. Więcej istotnych klinicznie informacji uzyskano w łączonej fazie naczyniowej i wydalniczej, z oceną anatomiczną unaczynienia i miąższu nerek włącznie. Dawka promieniowania jonizującego była podobna lub nawet niższa w porównaniu do pozostałych protokołów badania.

**Wnioski:**

Jednofazowa Uro-TK wykorzystująca podzielony bolus środka kontrastowego jest nowym sposobem obniżenia dawki promieniowania jonizującego w diagnostyce wad wrodzonych układu moczowego u dzieci. Połączenie fazy naczyniowej i wydalniczej okazało się bardzo efektywną metodą oceny anatomicznej wady, zwłaszcza przed planowaną interwencją chirurgiczną.

## Introduction

As considered by the ESPR and ESUR groups, the only indications for CT Urography (CTU) in children include: suspected nephrolithiasis, renal trauma, some tumors and complicated infections [1, 2]. MR Urography (MRU) has become a standard in other diseases or abnormalities of urinary tract in children. However, there is a role for CTU in case MRU is not available [2, 3].

CTU can be routinely performed in children with suspected congenital anomalies of kidneys and urinary tract (CAKUT) [4], especially before planned surgical intervention. Shorter time of examination and no need or much shorter time of sedation, in comparison to MRU, are an advantage [3, 5]. Low-dose protocols can help to achieve ALARA (as low as reasonably achievable) principle [4].

Split-bolus technique has already been described as a method of radiation dose reduction in CTU [1, 2, 6, 7]. Contrast medium (CM) is divided in two portions and administered with a several minutes interval. The scan is performed soon after the second dose. This allows to obtain two phases during one scan, with sparing the radiation dose. The diagnostic value of both phases is maintained. The study can be performed in different protocols, even as a triple-bolus one-phase CTU [3, 7, 8]. They differ by contrast volume per portion and by the intervals between portions.

CTU is considered as reliable technique in diagnosis of CAKUT [9, 10]. However − to the best of our knowledge – this is the first publication describing one-phase split-bolus low dose CTU technique in diagnosis of CAKUT in children. We present our experience with LD-CTU protocol, which was developed during the last 6 years in our Department.

## Objective

To present the protocol and determine the role of LD-CTU in diagnostic imaging of CAKUT in children.

## Material and methods

During the last 6 years (2011-2016) we have performed 276 CTUs in 249 children. This number includes standard indications, mentioned above (renal trauma, tumors, complicated infections and nephrolithiasis), but also suspected CAKUT.

CTUs were performed with two different multidetector CT scanners (64-row Philips Brilliance and 320-row Toshiba Aquilion ONE).

**Fig. 1:**
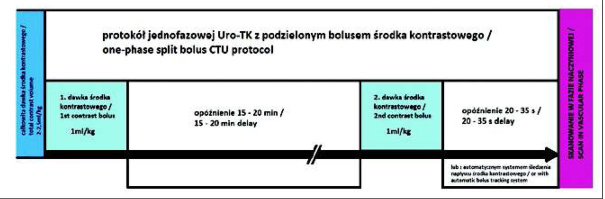
Scheme of the one-phase vascular-excretory split-bolus CTU technique in children. Total contrast volume (2-2.5 ml/kg) is divided half-and-half for vascular and excretory phases. Standard time interval between contrast bolus injections is 15-20 minutes, and the scan is performed 20-35 s after second dose of CM (vascular phase). An automatic bolus tracking system can also be used. Ryc. 1. Schemat jednofazowej Uro-TK z podzielonym bolusem środka kontrastowego, łączącej fazę naczyniową i wydalniczą. Całkowita dawka środka kontrastowego (2-2,5 ml/kg) została podzielona po połowie dla obu faz. Standardowa przerwa pomiędzy podaniem obu dawek środka kontrastowego wynosi 15-20 min, skanowanie wykonywane jest 20-35 s po podaniu drugiej dawki (w fazie naczyniowej). Automatyczny system śledzenia napływu środka kontrastowego może zostać wykorzystany do przeprowadzenia badania.

CTU was performed in 4 different protocols:

− Protocol 1: one-phase excretory CTU − performed 15-20 minutes after injection of CM in dose 1 ml/kg;
− Protocol 2: one-phase split-bolus CTU, with combination of parenchymal and excretory phases [6, 7] − 2-2,5 ml/kg of CM was divided half-and-half, standard time interval between CM bolus injections was 15-20 minutes, and the scan was performed 40-60s after second dose of CM;
− Protocol 3: one-phase split-bolus CTU, with combination of vascular and excretory phases (fig. 1) − protocol was similar to no. 2, however, scan was performed 20-35s after second dose of CM bolus injection, so earlier than in parenchymal phase. An automated bolus tracking system was also used in several studies, with ROI placed in descending aorta at the level of the diaphragm.
− Protocol 4: excretory phase was performed as a part of a multiphase study.

Whenever possible, low acquisition parameters were used, to meet ESPR and ESUR criteria [1, 2] and our guidelines described in previous publications [4].

CTU protocol evolved in time, and in some cases it could slightly differ from mentioned above - this concerned especially doses of CM per phase and time intervals between CM bolus injections (i.e. it could be elongated in some patients with severe hydronephrosis).

Examinations performed due to suspected CAKUT and follow-up studies performed after surgical repair were chosen to the final analysis − that is 226 CTUs in 205 patients (190 CTUs performed on Philips Brilliance, 36 – on Toshiba Aquilion ONE). In most cases, CTU was performed if no correlation was observed between the results of different imaging studies (especially ultrasonography and dynamic scintigraphy) or before qualification for surgical repair of the abnormality. Whenever possible, CTU was performed as a one-phase study.

## Results

In all study protocols, visualization of the urinary tracts was adequate to make the correct diagnosis and select an appropriate management method.

Indications for the CTUs in all study protocols are listed in table I. Most CTUs were performed only in excretory phase (Protocol 1 − 104 examinations), while 88 in split-bolus technique. This number contains parenchymal-excretory protocol (Protocol 2 − 33 CTUs) and vascular-excretory protocol (Protocol 3 – 55 CTUs). 34 examinations were performed as a part of a multiphase study (two or more phases, with at least one excretory phase) – Protocol 4.

**Table I:** Indications for 226 CTU examinations in 205 children. Tabela I. Wskazania do wykonania 226 badań Uro-TK u 205 dzieci.

Indication/*Wskazanie*	Protocol 1 *Protokół 1*	Protocol 2 *Protokół 2*	Protocol 3 *Protokół 3*	Protocol 4 *Protokół 4*	TOTAL *SUMA*
Hydronephrosis *Wodonercze*	46	9	38	12	105
Upper urinary tract duplication *Zdwojenie górnych dróg moczowych*	20	13	9	8	50
Megaureter *Moczowód olbrzymi*	23	4	4	5	36
Ureterocele *Ureterocele*	2	1	0	0	3
Post-operative follow-up (diagnosis of complications and/or assessment of outcomes) *Ocena pooperacyjna (diagnostyka powikłań i/lub efektów leczenia operacyjnego)*	10	4	2	5	21
Abnormalities of kidney structure, shape and location *Nieprawidłowości budowy, kształtu i lokalizacji nerek*	3	2	2	4	11
TOTAL *SUMA*	104	33	55	34	

In the group of 105 children with hydronephrosis, 62 were qualified for surgical treatment, while the remaining group was qualified for further observation. There were 15 patients with suspected crossing vessel as the cause of hydronephrosis (four patients in Protocol 1, eight patients in Protocol 3 and three patients in Protocol 4). Only Protocol 3 directly visualized the crossing vessel, while in Protocols 1 and 4 – there were only indirect signs, like compression of the ureter’s contour (none of 3 CTUs in Protocol 4 was performed with a vascular phase).

In 50 patients with suspected duplication of the urinary tract, the abnormality was ruled out in 12 children (four patients in Protocol 1, six patients in Protocol 2, one patient in Protocol 3 and one patient in Protocol 4). 24 patients were qualified to surgical repair (heminephrectomy). There were complications in 4 children, due to suspected post-operative urine leakage from the cut surface – preoperative CTUs were performed in Protocol 1 (two cases), Protocol 2 (one case) and Protocol 3 (one case). Urine leakage was confirmed in post-operative CTUs in 3 children (two examinations performed in Protocol 1, one examination in Protocol 4) – surgical revision was performed in all patients. One CTU, performed in Protocol 1, ruled out the urine leakage.

## Discussion

The role of CTU in children is different than in adults, where optimal distention and opacification of the collecting system, ureters and urinary bladder is essential for detection of urothelial cancer [7]. In diagnosis of CAKUT in children, it is crucial to visualize the anatomy of the abnormality, therefore MRU has become a method of choice [11, 12]. However, in many clinical situations LD-CTU could be performed instead of MRU.

Our analysis of the diagnostic accuracy of CTUs performed in different protocols showed that all of them provide adequate visualization of the urinary tract. However, there is a significant difference when the complete urinary system must be assessed, including vascular and renal anatomy.

One-phase excretory CTU (Protocol 1) is a standard procedure in CAKUT diagnosis [4]. However, it provides information only about collecting systems, ureters and urinary bladder, and in some cases additional scans in different phases are necessary. This will multiply radiation dose.

Split-bolus technique has been already described as a method of radiation dose reduction in CTU [1, 2, 6, 7]. There are several protocols, in CT or MR Urography, with different combinations of vascular, parenchymal and excretory phases [6, 7, 8], but - to the best of our knowledge − it was not described for diagnosis of CAKUT in children.

Combination of parenchymal and excretory phases (Protocol 2) in most analyzed cases did not bring more clinical information. Enhancement of renal parenchyma may reveal renal scars or focal lesions, however, this is not a main goal in suspected CAKUT. Renal scars will be evaluated in scintigraphy or MRU, while focal lesions are not routinely suspected in such patients. However, parenchymal enhancement can help to visualize general renal anatomy before planned surgical intervention.

**Fig. 2:**
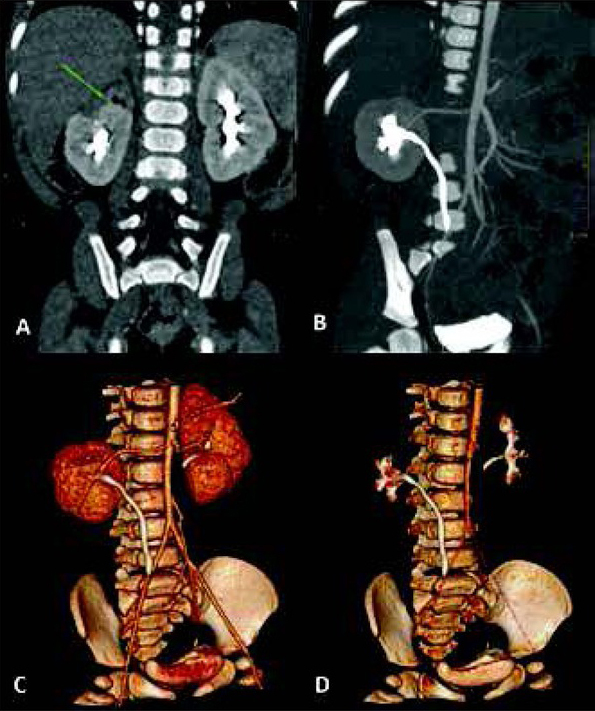
One-phase split-bolus CTU performed before planned surgical intervention in an 8-month-old girl with dys-plastic upper pole of duplicated right kidney. Coronal and volume reconstructions (A-D) show renal anatomy with dysplastic parenchyma (green arrow) and anatomy of vessels and lower collecting system. Ryc. 2. Jednofazowa Uro-TK z podzielonym bolusem środka kontrastowego wykonana u 8-miesięcznej dziewczynki przed planowaną operacją heminefrektomii z powodu dysplazji górnego układu zdwojonej nerki prawej. Rekonstrukcje czołowe i objętościowe (A-D) przedstawiają anatomię nerki z dysplastycznym miąższem (zielona strzałka) oraz anatomię naczyń i dolnego układu zbiorczego nerki.

**Fig. 3:**
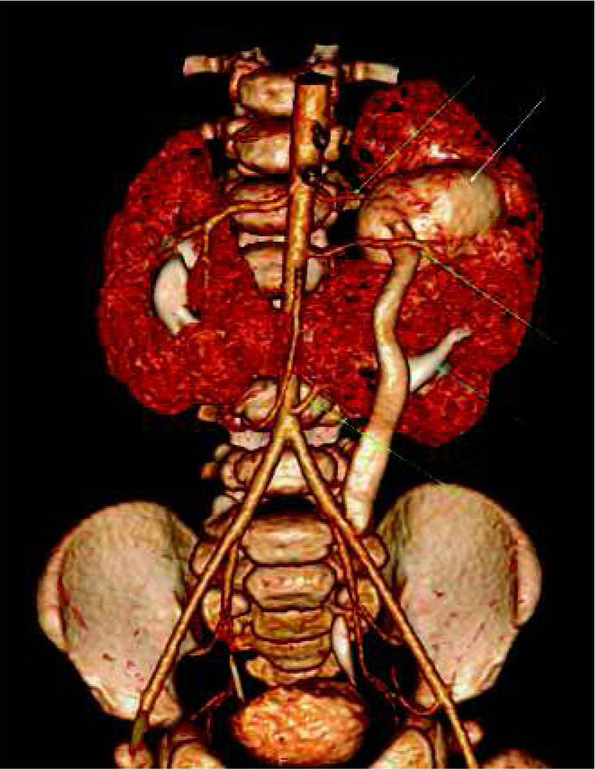
Volume reconstruction after one-phase split-bolus CTU performed in a 7-month-old boy with duplication of the collecting system in the left part of the horseshoe kidney (white and blue arrows), with rotation and hydronephrosis of the upper system (white arrow) and a megaureter. There are three renal arteries on the left side (green arrows), and the middle one crosses and compresses proximal part of the left upper ureter. Ryc. 3. Rekonstrukcja objętościowa jednofazowej Uro-TK z podzielonym bolusem środka kontrastowego wykonanej u 7-miesięcznego chłopca ze zdwojeniem lewej części nerki podkowiastej (białe i niebieskie strzałki), z rotacją i wodonerczem górnego układu (biała strzałka) oraz moczowodem olbrzymim. Badanie wykazało obecność trzech tętnic nerkowych po stronie lewej (zielone strzałki), z których środkowa krzyżuje i uciska moczowód górnego układu.

Combination of vascular and excretory phases (Protocol 3) provides the most complex visualization of the urinary system. It allows to asses abnormalities of the collecting system, with additional information about renal vascularity and general renal anatomy. It appears very useful especially before planned surgical intervention, e.g. heminephrectomy in duplicated collecting systems, where renal vessels and borders between both parts of the kidney should be evaluated (fig. 2). Vascular phase in this technique is not a “pure” CT-angiography, and it allows to assess renal parenchyma as well - higher heart rates in children will early enhance renal parenchyma in cortico-medullary phase [13]. Also, this protocol allows to detect crossing vessels as the cause of hydronephrosis [14, 15]. In comparison to other protocols, Protocol 3 will directly visualize the additional renal artery crossing and compressing the proximal ureter (fig. 3).

Multiphase CTU (Protocol 4) exposes the patient to higher radiation doses (which are multiplied with every additional phase) and should not be routinely performed in children. Similar diagnostic accuracy can be obtained in one-phase studies.

In our material, implementation of both split-bolus techniques (Protocol 2 and Protocol 3) allowed to more accurately assess the kidney anatomy and it was a milestone in planning the surgery, as assessed by the urologists. Due to additional visualization of renal vessels, Protocol 3 (combination of vascular and excretory phases) appears as more effective split-bolus CTU technique. It must be emphasized, however, that study protocol must suit the diagnostic problem. In case of suspected urine leakage after surgery (e.g. heminephrectomy), we suggest to perform the follow-up post-operative CTU in a single excretory phase (Protocol 1), as parenchymal enhancement in split-bolus technique may obscure the leakage.

**Fig. 4:**
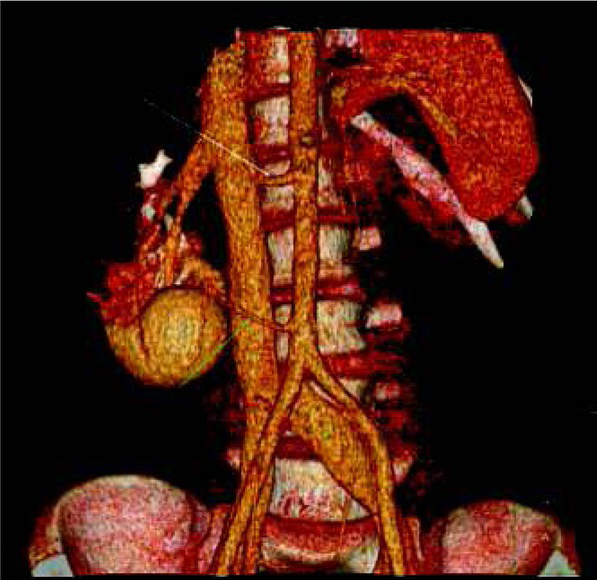
Volume reconstruction of CT performed in a 14-year-old boy with agenesis of the left kidney, after traumatic injury of the right kidney. One-phase split-bolus CT shows two renal arteries (arrows) with amputation of the upper artery (white arrow) and no enhancement of corresponding upper and middle part of the kidney. There were no signs of injury of the collecting system. Ryc. 4. Rekonstrukcja objętościowa TK wykonanej u 14-letniego chłopca z agenezją nerki lewej, po urazie nerki prawej. Jednofazowa TK z podzielonym bolusem środka kontrastowego wykazała obecność dwóch tętnic nerkowych (strzałki) oraz brak zakontrastowania dystalnego odcinka górnej tętnicy (biała strzałka) i odpowiadającej jej środkowej i górnej części miąższu nerki. Badanie nie wykazało zmian urazowych układu zbiorczego nerki.

Our analysis showed that one-phase split-bolus LD-CTU protocol is a very effective and dose sparing technique in diagnosis of CAKUT in children. Additionally, it can be adapted to the standard indications agreed by the ESPR and ESUR groups [1, 2]. In renal trauma, not only renal parenchyma can be assessed during one scan, but also vessels and collecting system (fig. 4) [1, 2, 7]. This will reduce the need of multiple scanning, especially that initially the site of injury may be not known. In imaging diagnosis of tumors and complicated infections, parenchymal phase is considered suffcient in most cases [1, 2]. However, in some specific situations additional scans can be required. The primary role of imaging in renal tumors in children is the preoperative assessment and evaluation for metastatic disease [16], including venous invasion. During diagnosis of complicated pyelonephritis, the site of obstruction or congenital abnormality which predisposes to infection [5] can be revealed with one-phase split bolus technique.

There were limitations to our study. We haven’t compared directly the image quality and dose reduction between the study protocols, or differences in diagnostic accuracy between both CT scanners. The main goal was to present advantages of one-phase split-bolus CTU technique. In comparison to multiphase studies, where the radiation dose multiplies with every additional phase, implementation of one-phase CTU protocol allowed to significantly reduce radiation dose. Furthermore, in comparison to different CTU protocols, diagnostic accuracy increased significantly when the one-phase split-bolus CTU with combination of vascular and excretory phases was implemented. This protocol is universal and can be adapted to every CT scanner, nevertheless, there are still many issues for further investigations, especially with protocol optimization (e.g. evaluation of image quality in lower dose examinations). Also, the optimal use of CM should be evaluated, especially in terms of CM volume and time interval between CM bolus injections. From our experience, it is recommended to use at least 2 ml/kg of total dose of CM, divided half-and-half for vascular and excretory phases. Lower doses, like standard 1 - 1,5 ml/kg divided for both phases, could be not suffcient to appropriately enhance renal vessels, parenchyma and collecting system, especially in case of hydronephrosis.

## Conclusions

Low-dose one-phase split-bolus CTU protocol is a reliable technique to reduce radiation dose in children. Combination of vascular and excretory phases provides very precise picture of CAKUT, especially before planned surgical intervention. It can become a standard CTU protocol in children.

## References

[1] Damasio MB, Darge K, Riccabona M (2013). Multi-detector CT in the paediatric urinary tract. Eur J Radiol.

[2] Riccabona M, Avni FE, Dacher J-N, Damasio MB, Darge K, Lobo L, Ording-Muller LS, Papadopolou F, Willi U (2010). ESPR uroradiology task force and ESUR paediatric working group: imaging and procedural recommendations in paediatric uroradiology, part III. Minutes of the ESPR uroradiology task force minisymposium on intravenous urography, uro-CT and MR-urography in childhood. Pediatr Radiol.

[3] Darge K, Higgins M, Hwang TJ, Delgado J, Shukla A, Bellah R (2013). Magnetic resonance and computed tomography in pediatric urology: an imaging overview for current and future daily practice. Radiol Clin North Am.

[4] Bombiński P, Warchoł S, Brzewski M, Biejat A, Dudek-Warchoł T, Krzemień G, Szmigielska A. (2014). Lower-dose CT urography (CTU) with iterative reconstruction technique in children: initial experience and examination protocol. Pol J Radiol.

[5] Chung EM, Soderlund KA, Fagen KE (2017). Imaging of the Pediatric Urinary System. Radiol Clin N Am.

[6] Chow LC, Kwan SW, Olcott EW, Sommer G (2007). Split-bolus MDCT urography with synchronous nephrographic and excretory phase enhancement. AJR Am J Roentgenol.

[7] Van Der Molen AJ, Cowan NC, Mueller-Lisse UG, Nolte-Ernsting CC, Takahashi S, Cohan RH. (2008). CT urography: definition, indications and techniques: a guideline for clinical practice. Eur Radiol.

[8] Kekelidze M, Dwarkasing RS, Dijkshoorn ML, Sikorska K, Verhagen PC, Krestin GP (2010). Kidney and urinary tract imaging: triple-bolus multidetector CT urography as a one-stop shop−protocol design, opacification, and image quality analysis. Radiology.

[9] Venkateswar R Surabhi, Christine O. Menias, Verghese George, Eduardo Matta, Ravi K. Kaza, Joseph Hasapes (2015). MDCT and MR Urogram Spectrum of Congenital Anomalies of the Kidney and Urinary Tract Diagnosed in Adulthood. American Journal of Roentgenology.

[10] Ramanathan S, Kumar D, Khanna M, Al Heidous M, Sheikh A, Virmani V, Palaniappan Y. (2016). Multi-modality imaging review of congenital abnormalities of kidney and upper urinary tract. World Journal of Radiology.

[11] Battal B, Kocaoglu M, Akgun V, Ince S, Gok F, Tasar M (2015). Split-bolus MR urography: synchronous visualization of obstructing vessels and collecting system in children. Diagn Interv Radiol.

[12] Lipson JA, Coakley FV, Baskin LS, Yeh BM (2008). Subtle renal duplication as an unrecognized cause of childhood incontinence: Diagnosis by magnetic resonance urography. J Pediatr Urol.

[13] Dahlman P, van der Molen AJ, Magnusson M, Magnusson A. (2012). How much dose can be saved in three-phase CT urography? A combination of normal-dose corticomedullary phase with low-dose unenhanced and excretory phases. AJR Am J Roentgenol.

[14] Cain MP, Rink RC, Thomas AC, Austin PF, Kaefer M, Casale AJ (2001). Symptomatic ureteropelvic junction obstruction in children in the era of prenatal sonography: is there a higher incidence of crossing vessels?. Urology.

[15] Weiss DA, Kadakia S, Kurzweil R, Srinivasan AK, Darge K, Shukla AR (2015). Detection of crossing vessels in pediatric ureteropelvic junction obstruction: clinical patterns and imaging findings. J Pediatr Urol.

[16] Chung EM, Graeber AR, Conran RM (2016). Renal tumors of childhood: radiologic-pathologic correlation part 1. The 1st decade: from the radiologic pathology archives. Radiographics.

